# Overexpression of mGluR7 in the Prefrontal Cortex Attenuates Autistic Behaviors in Mice

**DOI:** 10.3389/fncel.2021.689611

**Published:** 2021-07-15

**Authors:** Xiaona Wang, Chao Gao, Yaodong Zhang, Shunan Hu, Yidan Qiao, Zhengqin Zhao, Lingshan Gou, Jijun Song, Qi Wang

**Affiliations:** ^1^Department of Nuclear Medicine, Affiliated Hospital of Guangdong Medical College, Zhanjiang, China; ^2^Department of Rehabilitation, Children’s Hospital Affiliated to Zhengzhou University, Zhengzhou, China; ^3^Department of Pathology, Children’s Hospital Affiliated to Zhengzhou University, Zhengzhou, China; ^4^Center for Genetic Medicine, Xuzhou Maternity and Child Health Care Hospital, Xuzhou, China; ^5^Henan Infectious Disease Hospital, The Sixth People’s Hospital of Zhengzhou, Zhengzhou, China; ^6^Department of Histology and Embryology, Guizhou Medical University, Guizhou, China

**Keywords:** autism spectrum disorder, metabotropic glutamate receptor 7, prefrontal cortex, action potential, mouse models

## Abstract

Autism spectrum disorder (ASD) is associated with a range of abnormalities pertaining to socialization, communication, repetitive behaviors, and restricted interests. Owing to its complexity, the etiology of ASD remains incompletely understood. The presynaptic G protein-coupled glutamate receptor metabotropic glutamate receptor 7 (mGluR7) is known to be essential for synaptic transmission and is also tightly linked with ASD incidence. Herein, we report that prefrontal cortex (PFC) mGluR7 protein levels were decreased in C57BL/6J mice exposed to valproic acid (VPA) and BTBR T^+^ Itpr3^tf^/J mice. The overexpression of mGluR7 in the PFC of these mice using a lentiviral vector was sufficient to reduce the severity of ASD-like behavioral patterns such that animals exhibited decreases in abnormal social interactions and communication, anxiety-like, and stereotyped/repetitive behaviors. Intriguingly, patch-clamp recordings revealed that the overexpression of mGluR7 suppressed neuronal excitability by inhibiting action potential discharge frequencies, together with enhanced action potential threshold and increased rheobase. These data offer a scientific basis for the additional study of mGluR7 as a promising therapeutic target in ASD and related neurodevelopmental disorders.

## Introduction

The term autism spectrum disorder (ASD) is used to describe a series of heritable neurodevelopmental disorders with a heterozygous presentation that can present in the form of abnormal communication, social interaction, repetitive behaviors, and restricted interests. While ASD etiology remains incompletely understood, glutamate-mediated neurotransmission and synapse dysfunctions are both thought to be closely related to the onset of this disorder (Al Otaish et al., 2018; Wang et al., 2018c; Khalifa et al., 2019). Metabotropic glutamate receptors are of particular importance in the context of ASD development, and function as mediators of excitatory synaptic neurotransmitter signaling and plasticity in the brains of mammals (Aguilar Valles et al., 2015; Wang et al., 2016; Edfawy et al., 2019; Salpietro et al., 2019).

Five isoforms of metabotropic glutamate receptor 7 (mGluR7), which is encoded by *GRM7*, have been found to be expressed in the prefrontal cortex (PFC) in mammals (Millán et al., 2003; Wang et al., 2018a), and prior research has linked *GRM7* mutations to ASD incidence (Xia et al., 2015; Fisher et al., 2018; Yang et al., 2019). Indeed, a loss of mGluR7 expression in mouse model systems is sufficient to disrupt normal motor functions, associative learning, and social behavior-related phenotypes (Fisher et al., 2020). Similarly, mGluR7-I154T mutant animals exhibited reduced mGluR7 expression, deficits in contextual fear learning, and seizures (Fisher et al., 2021). As a mediator of neuronal excitability, mGluR7 has been linked to several neuropsychiatric disorders (Yang and Pan, 2013; Liu et al., 2015; Takarae and Sweeney, 2017). Increased neuronal excitability is thought to be critical for many of the behavioral and functional phenotypes characteristic of ASD (Takarae and Sweeney, 2017; Lo and Erzurumlu, 2018; Hajisoltani et al., 2019). However, the specific mechanistic role for mGluR7 as a regulator of neuronal excitability and ASD-related behavioral abnormalities remains to be fully clarified.

Herein, we sought to evaluate the importance of mGluR7 in the context of aberrant ASD-related behaviors. To that end, we assessed mGluR7 expression in a sodium valproic acid (VPA)-exposed and BTBRT^+^ Itpr3^tf^/J (BTBR) mice. We then overexpressed mGluR7 in the PFC in these two different murine models of ASD to examine the impact of this protein on neuronal excitability and behaviors. These analyses revealed mGluR7 downregulation in experimental mice such that the overexpression of this protein was able to attenuate neuronal excitability, which contributes to lessening the severity of ASD-related phenotypes. Overall, these findings suggest that mGluR7 overexpression may represent a viable approach to treating ASD.

## Materials and Methods

### Animals

The animal experiment ethics committee of Zhengzhou University approved all animal studies conducted herein. C57BL/6J and BTBR mice (8 weeks old, 24–25 g, Jackson Laboratory, ME, USA) were housed in cages under standard laboratory conditions (12 h dark/light cycle) with free food and water access.

### Drug Treatment and Experimental Grouping

As we reported previously (Wang et al., 2018b), C57BL/6J mice were mated and examined for the presence of a vaginal plug, which was used to define embryonic day 1 (E1). On E13, pregnant female mice were intraperitoneally injected with 600 mg/kg of VPA (Sigma Aldrich, MO, USA) in 0.9% saline, while control animals were instead injected with an equivalent volume of saline solution. On postnatal day 21, pups were weaned and transferred to cages (*n* = 3/cage). C57BL/6J mice are commonly used as controls for BTBR mice in ASD-related studies. Only male offspring were used in subsequent experiments.

On postnatal day 35, sodium pentobarbital was used to deeply anesthetize mice, which were subsequently decapitated. The PFC was rapidly dissected from mice between approximately + 1.85 and + 1.15 mm from the bregma (Franklin and Paxinos, 2007). Tissues were then collected for downstream experiments.

### Western Blotting

A whole protein extraction kit (Keygen Biotech, Nanjing, China) was used to extract total protein from PFC tissue samples. Lysates were heated to 98°C for 5 min after calculating the protein concentrations therein with an enhanced BCA kit. Protein samples (30 μg each) were then separated *via* SDS-PAGE and transferred onto PVDF membranes (Millipore, USA) with an electrophoretic transfer system (Bio-Rad Laboratories, USA). Blots were then probed overnight with anti-mGluR7 (1:1,000, rabbit, MyBioSource, CA, USA), and anti-β-actin (1:1,000, mouse, Beijing Biotech Co., Ltd, Beijing, China), at 4°C. After probing with secondary horseradish peroxidase-conjugated goat anti-rabbit/mouse IgG (1:1,000; ZSGB-Bio, Beijing, China), a super-enhanced chemiluminescence reagent (Pierce, IL, USA) was used to detect protein bands that were imaged with a luminescent image analysis system (Bio-Rad Laboratories). The ImageJ software was then used for densitometric quantification of the detected bands.

### Lentiviral Injection Into the Prefrontal Cortex

A lentivirus encoding mGluR7 (pLenti-CaMKIIa-mGluR7-EGFP; 3 × 10^8^ Tu/ml) was purchased from Shanghai Biotechnology Co., Ltd., with a virus encoding EGFP (LV-GFP; 3 × 10^8^ Tu/ml) serving as a control. The vectors promote EGFP expression in the mature post-mitotic cortical excitatory glutamatergic neurons (Dittgen et al., 2004; Parr-Brownlie et al., 2015).

Lentiviral injection into the PFC was conducted using a slightly modified version of a previously published protocol (Wang et al., 2020). Briefly, mice were intraperitoneally injected with pentobarbital sodium (80 mg/kg) and transferred to a stereotaxic apparatus. A glass pipette was then used to infuse 2 μl LV-mGluR7 or LV-GFP at 0.2 μl/min unilaterally in the PFC (anterior-posterior 1.8 mm, medial-lateral ±0.35 mm, and dorsal-ventral 2.0 mm). The needle was then allowed to rest in the injection site for 10 min following the completion of the injection to ensure that all viral particles remained within the PFC.

Five days after the injection of LV vectors, mice were randomly selected from each experimental group and used for immunofluorescence analyses. Western blotting was used to evaluate the efficiency of AAV vector interventions, which were also quantified at 14 and 35 days after injection.

### Immunohistochemistry

After animals were sacrificed, brain tissue samples were fixed with 4% paraformaldehyde, dehydrated using 30% sucrose, and embedded with optimal cutting temperature compound (Sakura Finetek, Tokyo, Japan). Sagittal 40 μm thick sections were then prepared with a freezing-sliding Microtome (Leica, Germany) as in previous reports (Wang et al., 2018b). These sections were then permeablized using phosphate buffer containing 0.25% Triton X-100 prior to overnight incubation with anti-GFP (1:1,000, Invitrogen, CA, USA) in blocking solution at 4°C. An Alexa Fluor 488-conjugated donkey anti-goat IgG secondary antibody (Invitrogen) was then used to probe samples for 2 h, after which immunoreactive sections were assessed using a Zeiss 900 laser scanning confocal microscope (Carl Zeiss, Jena, Germany).

Further, quantification of GFP-expressing cells in the PFC of treated mice was performed using the optical fractionator sampling method, as we previously described (Wang et al., 2018b). In brief, six sections of each mouse (*n* = 6) at a fixed 160 μm interval were systematically randomly sampled from the PFC. The size of the counting frame was 30 × 30 μm. The dissector height was 12 μm. Number (N) of GFP-positive cells was calculated using the optical fractionator where *N* = ΣQ * t/h * 1/asf * 1/ssf, as in West et al. (1991).

### Behavioral Analyses

At 3 weeks following lentiviral injection, mice underwent social interaction, open field, elevated plus maze, and marble burying tests on four consecutive days. Electrophysiological recordings were made 24 h following the end of the marble burying task. The ANY-Maze software (Stoelting Co., IL, USA) was used for all behavioral recordings.

### Sociability and Social Novelty Testing

Sociability testing was conducted using a 3-chamber black plastic box apparatus using a slightly modified version of the previously published protocol (Kim et al., 2016). The 3-chamber box (45 × 30 × 20 cm) contained dividing walls with 10 × 12 cm openings allowing access into each compartment. Test animals were sex-and strain-matched with unfamiliar mice with which they had not had any prior contact. At the start of the test, a selected mouse was placed in the empty central chamber, with the selected unfamiliar mouse being placed in a wire cage (12 cm diameter) in the left chamber, and an empty wire cage (novel object) being placed in the right compartment. Following a 5 min acclimation period, sociability tests were conducted for 10 min.

For social novelty preference tests, a different novel animal was positioned within the wire cage in the right chamber, and the study subject was returned to the empty central chamber. Animal movements during the following 10 min were then monitored, with animals having free access to all three chambers.

### Open Field Test

Open field tests were conducted as in prior reports (Wang et al., 2020). Briefly, mice were dropped into the central region of a 48 cm (W) × 52 cm (L) × 40 cm (H) chamber, and both the total distance traveled and the time spent in the central region (15 × 15 cm) over a 10 min period were quantified.

### Elevated Plus Maze Test

Mice were positioned within a standard elevated plus-shaped maze apparatus composed of two opposing open arms and two arms that were enclosed (60 × 5 × 30 cm) for 5 min. Each mouse was placed on the center region, and both the total time traveled and the time spent in open arms was measured.

### Marble Burying Assay

A marble burying test was employed to test for stereotyped behaviors in ASD model rats (Vuillermot et al., 2017). Briefly, a 6 cm layer of chipped cedar wood bedding was applied to the bottom of clean cages (30 × 18 × 16 cm). Mice were then added to cages for 15 min, after which 15 colored glass marbles (15 mm in diameter) were added to the cage, with equal spacing between marbles. The number of marbles that were buried (>67% covered in bedding) after 10 min was counted, with digging being defined as coordinated limb movement that displaced the bedding.

### Patch-Clamp Recordings

Brain slices were collected from adult C57BL/6J male mice as discussed previously (Wang et al., 2020). Briefly, sodium pentobarbital (1%) was used to deeply anesthetize mice, after which the brain was rapidly collected and transferred into the ice-cold cutting artificial cerebrospinal fluid (ACSF) containing (in mm): 25 D-glucose, 110 choline chloride, 25 NaHCO_3_, 7 MgSO_4_, 1.25 NaH_2_PO_4_, 2.5 KCl, 0.5 CaCl_2_, 11.6 sodium ascorbate, and 3.1 sodium pyruvate. A Campden-5000 tissue slicer (Campden, England) was used to prepare 300 μm coronal brain tissue sections in chilled cutting solution. Those slices containing the PFC were warmed for 30 min at 36°C with ACSF containing (in mm): 25 D-glucose, 127 NaCl, 2.5 KCl, 25 NaHCO_3_, 1.25 NaH_2_PO_4_, 2 CaCl_2,_ and 1 MgCl_2_. Slices were then placed in a Nikon microscope (BX51WI) recording chamber and immersed in oxygenated ACSF (2–3 ml/min) at room temperature. Recording pipettes (3–5 MΩ) were filled with an internal solution containing (in mm): 10 NaCl, 128 potassium gluconate, 4 Na_2_ATP, 10 HEPES, 2 MgCl_2_, 0.5 EGTA and 0.4 Na_3_GTP. These solutions were equilibrated using 95% O_2_/5% CO_2_ prior to use. Recordings of neuronal excitability were made in current-clamp mode, with pyramidal cells being rejected if they exhibited a resting membrane potential >+50 mv or a spike amplitude of <60 mv. Action potential frequencies were measured in response to repeated 4 s duration current steps (10 pA/step). The parameters measured and calculated in this study also included the action potential threshold and current threshold (rheobase). The calculation of these parameters has been previously described (Dai et al., 2009). A HEKA EPC10 double patch-clamp amplifier (HEKA, Lambrecht, Germany) was used for data acquisition, with a sampling rate of 10 kHz and 2 kHz filtering.

### Statistical Analysis

Data are means ± SEM and were analyzed using SPSS 20.0. For social behavior, anxiety, and repetitive behavioral parameters, data were analyzed *via* one-way ANOVAs followed by* post hoc* Tukey’s multiple comparison test. For electrophysiology experiments, data were analyzed *via* repeated-measures ANOVA. Otherwise, comparisons between the two groups were made *via* two-tailed Student’s *t*-tests. *p* < 0.05 was the significance threshold for this study.

## Results

### VPA-Exposed and BTBR Mice Exhibit Reduced mGluR7 Expression in the PFC

**Figure 1:**
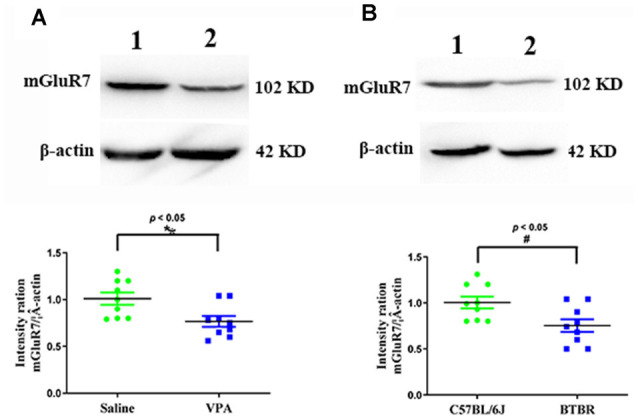
Quantitative analyses of mGluR7 expression in the prefrontal cortex (PFC) of valproic acid (VPA)-exposed and BTBR mice. Western blots and corresponding densitometric quantification assessing mGluR7 expression in the PFC of VPA-treated and BTBR mice. **(A)** Lane 1, saline-exposed controls; Lane 2, VPA-treated animals. **(B)** Lane 1, C57BL/6J group; Lane 2, BTBR group. Data are normalized to mGluR7 expression in control animals (100%). β-actin was a normalization control. *n* = 6 mice per group. *p < 0.05 vs. saline-treated controls. ^#^*p* < 0.05 vs. C57BL/6J group. The Student’s *t*-test assessed statistical significance. Data are means ± SEM.

We began by conducting Western blotting assays to assess mGluR7 protein levels in the PFC of VPA-treated and BTBR ASD model mice. We found that VPA exposure was linked with a significant reduction in mGluR7 protein expression in the PFC relative to levels in control animals (*p* < 0.05, Figure 1A). Consistently, mGluR7 levels were significantly lower in the BTBR mice relative to C57BL/6J controls (*p* < 0.05, Figure 1B). Together these results thus indicate that mGluR7 is downregulated in the PFC of VPA-exposed and BTBR mice. In addition, similar to mGluR7, mGluR2/3, mGluR4, and mGluR6 are coupled to Gi/o and negatively modulate adenylate cyclase (Jones et al., 2008) and are expressed in the rodent PFC (Tyszkiewicz et al., 2004; Wan et al., 2018; Fitzgerald et al., 2019). We found no apparent difference in the expression of mGluR2/3, mGluR4, and mGluR6 in VPA-treated mice compared to the control mice (*p* > 0.05, *p* > 0.05, *p* > 0.05). No differences were also observed in the mGluR2/3, mGluR4, and mGluR6 protein levels in BTBR mice compared with the C57BL6/J group (*p* > 0.05, *p* > 0.05, *p* > 0.05, Supplementary Figures 1A,B).

### Establishment of Mice Overexpressing mGluR7 in the PFC

**Figure 2:**
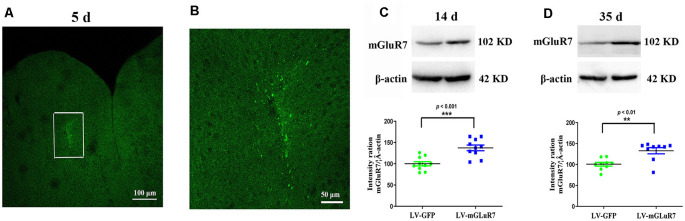
Confirmation of mGluR7 overexpression in a mouse model of autism spectrum disorder (ASD). **(A,B)** GFP expression in the PFC of VPA-exposed mice was detected on day 5 post-LV injection. The region outlined in white in **(A)** is shown at a higher magnification in **(B)**. **(C,D)** mGluR7 protein levels were measured in mGluR7-overexpressing VPA-treated mice on days 14 and 45 post-LV injections. Normalized intensity bands for mGluR7 are presented. Lane 1, LV-GFP-exposed group; Lane 2, LV-mGluR7-treated group. *n* = 6 mice in each group. ***p < 0.001, **p < 0.01. Student’s *t*-test. Results are shown as mean ± SEM.

To test the functional importance of mGluR7 in ASD, we began by overexpressing this protein in VPA-exposed mice *via* the injection of a mGluR7-encoding lentivirus into the PFC. On days 5 post-LV injection, GFP signal was evident in the PFC, consistent with successful sustained lentiviral delivery (Figures 2A,B). When the distribution of GFP-positive cells was assessed 5 days after LV-mGluR7 injection, we observed that number of GFP-expressing cells was (3.15 ± 0.12) × 10^4^.

Western blotting further confirmed that mGluR7 protein levels were significantly increased in VPA-treated mice that had been injected with LV-mGluR7 relative to LV-GFP-injected control mice at days 14 and 35 post-LV injections (*p* < 0.001, *p* < 0.01, Figures 2C,D). These results suggested that our lentiviral vector was able to successfully enhance mGluR7 protein expression in cells within the PFC *in vivo* Notably, VPA-treated mice overexpressing mGluR7 exhibited no significant difference in the expression of mGluR2/3, mGluR4, and mGluR6 compared to the mice that received LV-GFP (*p* > 0.05, *p* > 0.05, *p* > 0.05). No differences were detected in the mGluR2/3, mGluR4, and mGluR6 protein levels in BTBR mice overexpressing mGluR7 compared to the LV-GFP-treated mice (*p* > 0.05, *p* > 0.05, *p* > 0.05, Supplementary Figures 2A,B).

### mGluR7 Overexpression Alleviates Impaired Sociability and Preference for Social Novelty

**Figure 3:**
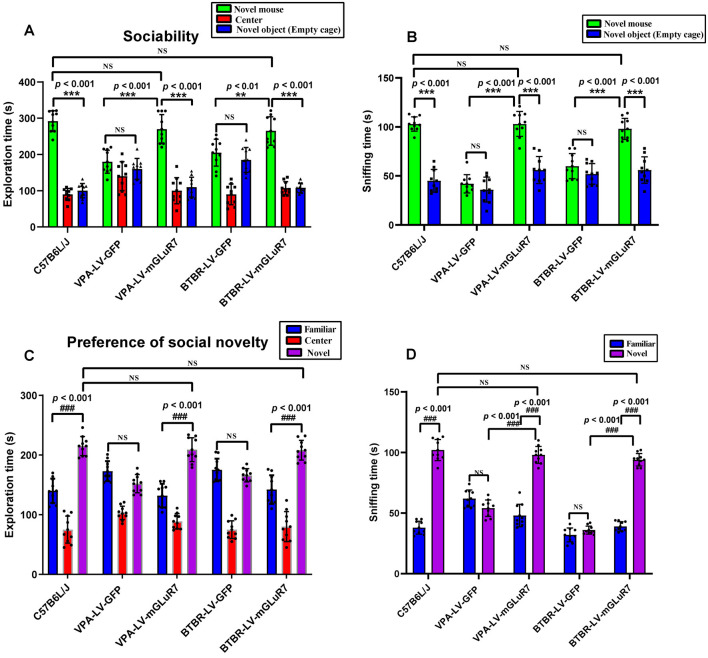
mGluR7 overexpression enhances murine social interactions in a three-chamber test. **(A)** In sociability tests analyzing the total time spent in the three chambers, C57B6L/J mice spent significantly more time in the chamber of the novel mouse relative to the novel object (*p* < 0.001). Administration of LV-GFP to VPA and BTBR mice was associated with these animals having spent equal amounts of time in the chambers containing the novel mouse and the novel object (*p* > 0.05; *p* > 0.05). In contrast, VPA-treated and BTBR mice overexpressing mGluR7 (LV-mGluR7-treated) exhibited an increase in the amount of time spent in the unfamiliar chamber relative to the empty chamber (*p* < 0.001; *p* < 0.001), consistent with normal sociability (*p* > 0.05; *p* > 0.05). VPA-treated and BTBR mice overexpressing mGluR7 spent significantly more time in the chamber of the novel mouse compared to LV-GFP-treated mice (*p* < 0.001, *p* < 0.01, respectively). **(B)** In sociability tests analyzing interaction time, C57B6L/J mice spent significantly more time sniffing the novel mouse relative to the novel object (*p* < 0.001). VPA-exposed and BTBR mice that had been injected with LV-GFP spent similar amounts of time interacting with the novel mouse and the novel object (*p* > 0.05; *p* > 0.05). Mice pretreated with LV-mGluR7 exhibited more interactions with the novel mouse relative to the novel object (*p* < 0.001; *p* < 0.001), consistent with normal sociability (*p* > 0.05; *p* > 0.05). The time spent with the novel mouse for VPA and BTBR mice differed significantly between LV-GFP and LV-mGluR7-treated animal subgroups (*p* < 0.001; *p* < 0.001). **(C)** In an assay testing for social novelty preferences, C57B6L/J mice spent significantly more time in the chamber of the novel mouse relative to the familiar mouse (*p* < 0.001). VPA-exposed and BTBR mice injected with LV-mGluR7 preferred exploring the chamber containing the novel mouse relative to familiar controls (*p* < 0.001; *p* < 0.001), consistent with normal social preference (*p* > 0.05; *p* > 0.05). In contrast, no significant difference in the time spent exploring the chamber containing the novel mouse was observed relative to the time spent exploring the familiar chamber for VPA-treated and BTBR mice treated with LV-GFP (*p* > 0.05; *p* > 0.05). **(D)** C57B6L/J mice spent significantly more time sniffing and engaging with the novel mouse relative to the familiar mouse (*p* < 0.001). In contrast, VPA-treated mice and BTBR mice that had been treated with LV-GFP spent equal amounts of time sniffing the novel mouse and the familiar mouse (*p* > 0.05; *p* > 0.05). VPA and BTBR mice treated with LV-mGluR7 exhibited significant increases in the time spent sniffing the novel mouse relative to controls (*p* < 0.001; *p* < 0.001), consistent with normal social preferences (*p* > 0.05; *p* > 0.05). The time spent by VPA-exposed and BTBR mice with the novel mouse differed significantly between the LV-GFP and LV-mGluR7-treated groups (*p* < 0.001; *p* < 0.001). ***p < 0.001 for novel mouse vs. novel object; ^###^*p* < 0.001 for novel side vs. familiar side. *n* = 10 animals per group. One-way ANOVA with Tukey’s *post hoc* test. Data are means ± SEM. **p < 0.01 for LV-mGluR7 vs. LV-GFP; “NS” indicating no statistical significance.

To examine the impacts of mGluR7 overexpression on behavioral changes in two mouse models of ASD, we next assessed their performance in tests designed to assess social behaviors, as impaired social interactions are a hallmark of ASD in humans (Scheeren et al., 2020). As shown in Figure 3A, the results of a *post hoc* analysis conducted using Tukey’s test indicated that C57B6L/J mice spent significantly more time in the chamber of the novel mouse relative to the novel object (*p* < 0.001). In sociability tests following VPA exposure, we determined that LV-mGluR7-treated mice spent more time in the chamber containing an unfamiliar mouse relative to the empty experimental chamber (*p* < 0.001), with this behavior being similar to normal sociability (*p* > 0.05). In contrast, this was not true for mice treated with LV-GFP (*p* > 0.05). LV-mGluR7-treated mice spent significantly more time in the chamber of the novel mouse compared to LV-GFP-treated mice (*p* < 0.001). BTBR mice treated with LV-GFP spent similar times in the chamber of the unfamiliar mouse relative to the novel object (*p* > 0.05). BTBR mice pretreated with LV-mGluR7 exhibited significant improvements in the time spent exploring the chamber containing the unfamiliar mouse relative to that containing the novel object (*p* < 0.001), consistent with normal sociability (*p* > 0.05). BTBR mice treated with LV-mGluR7 spent significantly more time in the chamber of the novel mouse compared with LV-GFP-treated mice (*p* < 0.01). These results are consistent with the amelioration of social behavior deficits.

The total time spent sniffing the novel mouse and novel object in each experimental condition was additionally measured for these experimental mice. As shown in Figure 3B, C57B6L/J mice spent markedly more time sniffing the novel mouse relative to the novel object (*p* < 0.001). In the VPA-induced ASD mouse model, LV-mGluR7-treated mice also spent more time closely interacting with the novel mice (*p* < 0.001), consistent with the normal social ability (*p* > 0.05). LV-GFP-treated mice did not exhibit any particular preference, consistent with a lack of interest in the novel mouse (*p* > 0.05). Among VPA mice, the time spent with the novel mouse was significantly different when comparing LV-GFP and LV-mGluR7-treated mice (*p* < 0.001). Additionally, BTBR mice pretreated with LV-GFP spent similar amounts of time interacting with novel mice and novel objects (*p* > 0.05), whereas BTBR mice pretreated with LV-mGluR7 spent significantly more time engaging in social interactions with novel mice relative to novel objects (*p* < 0.001), consistent with normal sociability (*p* > 0.05). The time spent with the novel mice in BTBR group mice differed significantly when comparing animals treated with LV-GFP and LV-mGluR7 (*p* < 0.001).

Furthermore, the time spent in individual compartments during social novelty preference testing was also measured (Figure 3C). C57B6L/J mice spent significantly more time in the chamber of the novel mouse relative to that of the familiar mouse (*p* < 0.001). In the VPA-induced ASD model group, however, there were no significant differences in the amount of time spent exploring these two chambers among LF-GFP treated animals (*p* > 0.05). VPA-exposed mice injected with LV-mGluR7 preferred exploring the chamber containing the unfamiliar mouse (*p* < 0.001), consistent with normal social preference (*p* > 0.05). Likewise, BTBR mouse pretreatment with LV-mGluR7 was associated with a significant increase in the preference for social novelty as measured by the time spent exploring the chamber containing novel mouse relative to that containing the familiar mouse (*p* < 0.001), consistent with normal social preference (*p* > 0.05). BTBR mice treated with LV-GFP, in contrast, did not exhibit any significant improvement in the time spent exploring the chamber containing the novel mouse relative to controls (*p* > 0.05).

As shown in Figure 3D, active interactions (based on sniffing behaviors) in the individual chambers in mice were additionally assessed. C57B6L/J mice spent significantly more time sniffing and engaging with a novel mouse relative to a familiar mouse (*p* < 0.001). In the VPA-induced mouse model of ASD, LV-mGluR7 injection was associated with mice having spent more time interacting with the novel mouse relative to the familiar mouse (*p* < 0.001), consistent with normal social preferences (*p* > 0.05). In contrast, LV-GFP-treated animals spent a similar amount of time sniffing the novel mouse and the familiar mouse (*p* > 0.05). The time spent with the novel mouse by VPA-treated mice was significantly different between the LV-GFP and LV-mGluR7-treated groups (*p* < 0.001). Similarly, LV-mGluR7 exposure in BTBR mice enhanced their social novelty preference (*p* < 0.001), consistent with normal social preferences (*p* > 0.05). However, LV-GFP failed to reverse these social novelty changes in BTBR mice (*p* > 0.05). The time spent with the novel mouse by BTBR mice differed significantly between LV-GFP and LV-mGluR7-treated mice (*p* < 0.001). These data were in line with the findings of the sociability test, suggesting that mGluR7 overexpression can remediate altered social novelty preferences in these experimental mice.

### mGluR7 Overexpression Ameliorates Anxiety-Like and Stereotyped/Repetitive Behaviors

**Figure 4:**
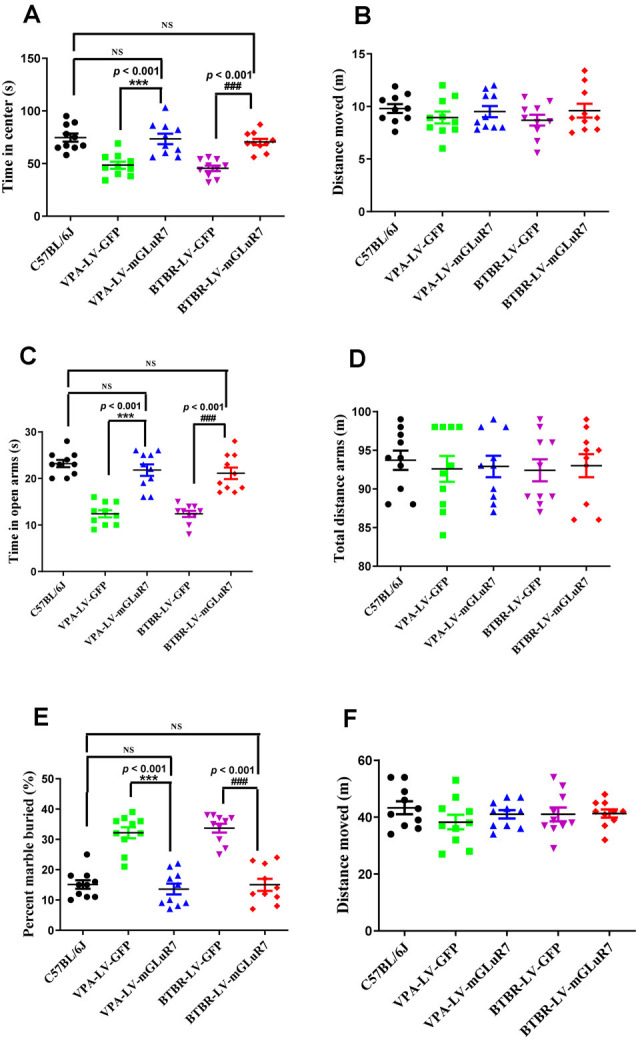
mGluR7 overexpression alleviates anxiety-like and stereotyped/repetitive behaviors. **(A,B)** The overexpression of mGluR7 in VPA-treated and BTBR mice increased the duration spent in the central area in an open field test to a similar extent as that observed in C57B6L/J mice (*p* < 0.05; *p* < 0.05), relative to VPA-exposed and BTBR animals (*F*_(2,27)_ = 38.23, *p* < 0.001; *F*_(2,27)_ = 38.01, *p* < 0.001), whereas total distance traveled was unchanged (*F*_(2,27)_ = 0.15, *p* > 0.05; *F*_(2,27)_ = 0.22, *p* > 0.05). **(C,D)** During elevated plus maze testing, overexpression of mGluR7 in these mice was similarly associated with increased time spent in the open arms relative to the VPA-treated and BTBR animals (*F*_(2,27)_ = 13.06, *p* < 0.001; *F*_(2,27)_ = 24.49, *p* < 0.001), to a similar extent as C57B6L/J mice (*p* > 0.05; *p* > 0.05). No significant difference in total distance traveled was observed (*F*_(2,27)_ = 0.73, *p* > 0.05; *F*_(2,27)_ = 1.17, *p* > 0.05). **(E,F)** In a marble burying test, mGluR7 overexpression was associated with a lower number of buried marbles relative to VPA-exposed and BTBR mice (*F*_(2,27)_ = 37.11, *p* < 0.001; *F*_(2,27)_ = 42.82, *p* < 0.001), to a similar extent as that observed in C57B6L/J animals (*p* > 0.05; *p* > 0.05). No significant differences in the total distance traveled were detected between groups of mGluR7-treated mice (*F*_(2,27)_ = 1.32, *p* > 0.05; *F*_(2,27)_ = 0.36, *p* > 0.05). *n* = 10 mice in each group. ***p < 0.001 vs. the VPA-exposed group; ^###^*p* < 0.001 vs. the BTBR group; NS indicates no statistical significance; One-way ANOVA. Data are means ± SEM.

To better understand whether LV-mGluR7 treatment was sufficient to alleviate anxiety-like behaviors in mouse models of ASD, as shown in Figures 4A,B, we next conducted open field tests in which the overexpression of mGluR7 in VPA-treated mice was associated with increased time spent in the center of the open field relative to LV-GFP treatment (*F*_(2,27)_ = 38.23, *p* < 0.001), similar to behaviors observed in C57B6L/J mice (*p* > 0.05). The total distance traveled in these groups was also comparable (*F*_(2,27)_ = 0.15, *p* > 0.05). LV-mGluR7 exposure significantly increased the amount of time spent in the center in BTBR mice relative to control animals (*F*_(2,27)_ = 38.01, *p* < 0.001), similar to what was observed in C57B6L/J mice (*p* > 0.05). The administration of LV-mGluR7 did not affect the total distance traveled by these animals (*F*_(2,27)_ = 0.22, *p* > 0.05).

Further, in the elevated plus maze test, treatment with LV-mGluR7 was associated with increased time spent in open arms relative to control treatment in VPA-exposed mice (*F*_(2,27)_ = 13.06, *p* < 0.001, Figure 4C), similar to what was observed in C57B6L/J mice (*p* > 0.05). The total distance traveled was comparable in these groups (*F*_(2,27)_ = 0.73, *p* > 0.05, Figure 4D). Likewise, LV-mGluR7 treatment results in a complementary increase in time spent in the open arms in BTBR animals relative to the LV-GFP-treated groups (*F*_(2,27)_ = 24.49, *p* < 0.001, Figure 4C), consistent with the results from C57B6L/J mice (*p* > 0.05). There were no significant differences between groups with respect to the total distance traveled (*F*_(2,27)_ = 1.17, *p* > 0.05, Figure 4D). A marble-burying experiment was employed to assess compulsive-like behaviors in our model mice (Wiebe and Nagpal, 2019; Hulbert et al., 2020). As shown in Figures 4E,F, mGluR7 overexpression in the VPA exposure model was associated with reduced numbers of marbles buried as compared to control lentiviral treatment (*F*_(2,27)_ = 37.11, *p* < 0.001), consistent with results from C57B6L/J animals (*p* > 0.05). No corresponding difference in the total distance moved in these groups was observed (*F*_(2,27)_ = 1.32, *p* > 0.05). Similarly, LV-mGluR7 exposure markedly decreased numbers of marbles buried when compared to LV-GFP alone (*F*_(2,27)_ = 42.82, *p* < 0.001), similar to results from C57B6L/J animals (*p* > 0.05). No significant differences in the total distance traveled were detected between groups (*F*_(2,27)_ = 0.36, *p* > 0.05). Together, our data from two animal models of ASD confirm that mGluR7 overexpression can alleviate core ASD-related phenotypes including anxiety and repetitive behaviors.

### mGluR7 Overexpression Suppresses Neuronal Excitability

**Figure 5:**
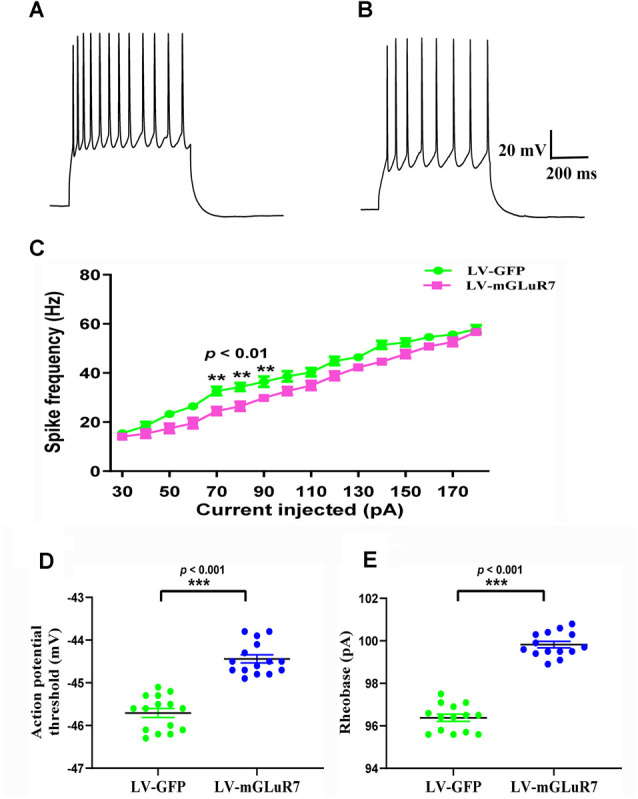
*In vitro* whole-cell recordings revealing the impact of mGluR7 on neuronal excitability in PFC slices. **(A,B)** Representative GFP-positive and mGluR7-expressing neurons traces corresponding to firing frequencies following the intracellular injection of step currents in mice exposed to VPA administered LV-GFP or LV-mGluR7. **(C)** Evoked spike rates vs. pyramidal cell current magnitudes in the PFC in the two treatment groups, exhibiting a significant reduction in mean firing frequency in LV-mGluR7-injected mice relative to controls (*F*_(1,28)_ = 11.023, *p* < 0.01; *F*_(1,28)_ = 10.058, *p* < 0.01; *F*_(1,28)_ = 8.129, *p* < 0.01). *n* = 8 animals per group. **p < 0.01; repeated measures ANOVA. Data are means ± SEM. **(D,E)** The action potential threshold of LV-mGluR7-exposed mice was significantly higher than those of LV-GFP-treated mice (*p* < 0.001). The rheobase of LV-mGluR7-exposed mice was significantly greater than those of controls (*p* < 0.001). *n* = 7 animals in each group. ***p < 0.001; Student’s *t*-test. Data are presented as means ± SEM.

Several studies to date have demonstrated that increased cortical excitability is associated with ASD-related behavioral phenotypes (Takarae and Sweeney, 2017; Lo and Erzurumlu, 2018; Hajisoltani et al., 2019). To examine the impact of mGluR7 overexpression on ASD-related neuronal excitability, we conducted patch-clamp recordings of PFC mGluR7-positive neurons in slices collected from the VPA-exposed rodent following lentiviral injection. These analyses revealed a significant reduction in action potential discharge frequency in slices from LV-mGluR7-treated mice exposure to VPA in response to depolarizing steps from 70–90 pA relative to slices from LV-GFP-treated mice (*F*_(1,28)_ = 11.023, *p* < 0.01; *F*_(1,28)_ = 10.058, *p* < 0.01; *F*_(1,28)_ = 8.129, *p* < 0.01, Figures 5A–C). The action potential threshold, the minimum voltage at which the action potential was generated, was markedly increased in LV-mGluR7-treated mice after exposure to VPA (*p* < 0.001, Figure 5D). Similar results were also obtained for rheobase. The rheobase, the minimal stimulation current to evoke action potentials, was significantly increased in LV-mGluR7-exposed mice compared to control mice (*p* < 0.001, Figure 5E). Action potential threshold and rheobase were essential parameters used to describe neuronal excitability. Consequently, these data were consistent with the animal ASD models wherein mGluR7 overexpression can suppress neuronal excitability.

## Discussion

The present study is the first to our knowledge to have demonstrated the downregulation of mGluR7 in the VPA-exposed and BTBR mice. We additionally found that the overexpression of mGluR7 using lentiviral vectors was sufficient to attenuate key ASD-related phenotypes in these mice, as evidenced by their enhanced social interactions, reduced anxiety-like traits, and decreased repetitive behaviors. Such mGluR7 overexpression additionally suppressed neuronal excitability *via* the inhibition of action potential discharge frequencies, accompanied by increases in action potential threshold and rheobase.

Herein, we found that mGluR7 expression was reduced in the PFC of VPA-exposed and BTBR mice. The heterozygous deletion of *GRM7* has previously been detected in patients with ASD, with affected patients exhibiting impaired social interactions, cognitive and language deficits, hyperactivity, and stereotyped behaviors (Gai et al., 2012; Liu et al., 2015). Decreased mGluR7 expression is thus likely to play a role in ASD-related behavioral impairment.

Hallmark symptoms of ASD include impairment in social behaviors (Meyza and Blanchard, 2017). Numerous reports have demonstrated that impaired sociability and preference for social novelty in the animal model of ASD induced by prenatal exposure to VPA (Baronio et al., 2015; Kim et al., 2016). Similarly, BTBR mice display impairment in sociability (Gould et al., 2012; Schwartzer et al., 2013, 2017) and preference for social novelty (Keum et al., 2016). In the current study, our experiments indicated that VPA-exposed and BTBR mice treated with LV-mGluR7 showed marked improvements in social behavior and social sniffing behavior equal to that observed in the social C57B6L/J strain. Overexpression of LV-mGluR7 also showed improvements in social engagement and preference for a social stimulus in the mice model of ASD. These data suggest that mGluR7 overexpression improved key ASD-related symptoms, including enhancing their reciprocal social interaction and communication.

Individuals with ASD are at an additional risk for developing anxiety (Perihan et al., 2020). A growing body of studies has supported the notion that mGluR7 has a dominant role in anxiety role (Kotlinska et al., 2019; O’Connor et al., 2019). Data with AMN082 would suggest the use of mGluR7 receptor agonists for treating anxiety (Spooren et al., 2010; Cieślik et al., 2018). Anxiety level is significantly higher in VPA-injected and BTBR mice (Gao et al., 2019; Queen et al., 2020). In agreement with previous results, we found overexpression of mGluR7 alleviates their anxiety-like traits.

Stereotype and behavior rigidity are widely known as core and defining features of ASD (Gilbert and O’Connor, 2020). Many lines of evidence have shown that VPA-treated and BTBR mice exhibited the increase in marble burying (Amodeo et al., 2012, 2014; Baronio et al., 2015). Consistent with the previous findings, we show that LV-mGluR7 overexpression restored the normal levels of marble-burying in experimental animals. Combined, these results indicate LV-mGluR7 treatment ameliorated repetitive/stereotyped behaviors.

While the mechanisms whereby mGluR7 overexpression can attenuate the key ASD-related behavioral phenotypes in these mice remain to be clarified, our data suggest that this beneficial activity is linked to the ability of this receptor to suppress neuronal excitability. We found that mGluR7 overexpression was associated with reduced PFC action potential discharge frequencies in slices from VPA-treated mice. Overexpression of mGluR7 also increased the stimulus threshold for action potential firing and the current required to evoke action potentials (rheobase). These changes reduced mGluR7-containing neurons’ excitability. Consistent with such models, increased cortical excitability is evident at the genetic, neuronal, and behavioral levels in ASD, and is closely linked to ASD-related aberrant phenotypes (Takarae and Sweeney, 2017). Indeed, when PFC mGluR7 was overexpressed in our ASD model mice, we found that it suppressed core ASD-related phenotypes through a mechanism directly associated with the suppression of such neuronal excitability. Convergent evidence implicates that excitability abnormalities within the PFC are thought to be linked to ASD-associated neuropathology (Spratt et al., 2019; Kelly et al., 2020; Sacai et al., 2020). Moreover, the PFC is connected with other cortical and sub-cortical regions of the brain including the hippocampus and amygdala involved in regulating behavioral aberrations of ASD. Future research to identify potent tool compounds capable of modulating mGluR7 function in corticolimbic circuits may thus hold promise as a means of pharmacologically treating behavioral phenotypes associated with ASD.

Summing up, the present data indicate that VPA-exposed and BTBR mice exhibit decreased mGluR7 overexpression. Lentivirus-mediated overexpression of mGluR7 in these mice can suppress neuronal excitability, and thereby ameliorate core ASD-related phenotypes. These results suggest that mGluR7 can modulate a diverse array of phenotypes overlapping with human symptoms of ASD, indicating that targeting this receptor protein may represent a valuable approach to treating relevant behavioral symptoms in affected individuals.

## Data Availability Statement

The original contributions presented in the study are included in the article/Supplementary Material, further inquiries can be directed to the corresponding author.

## Ethics Statement

The animal study was reviewed and approved by Compliance with Ethical Standards Animal treatment and maintenance were performed in accordance with the Principle of Laboratory Animal Care (NIH publication No. 85-23, revised 1985) and were approved by the Animal Care and Use Committee of the Zhengzhou University. Written informed consent was obtained from the owners for the participation of their animals in this study.

## Author Contributions

XW and CG conceived and designed research. YQ and LG performed the experiments. SH, JS, YZ, and ZZ analyzed the data. XW and QW prepared the article and critically revised the manuscript. All authors contributed to the article and approved the submitted version.

## Conflict of Interest

The authors declare that the research was conducted in the absence of any commercial or financial relationships that could be construed as a potential conflict of interest.
