# Corrigendum: Overexpression of mGluR7 in the Prefrontal Cortex Attenuates Autistic Behaviors in Mice

**DOI:** 10.3389/fncel.2021.747696

**Published:** 2021-08-26

**Authors:** Xiaona Wang, Chao Gao, Yaodong Zhang, Shunan Hu, Yidan Qiao, Zhengqin Zhao, Lingshan Gou, Jijun Song, Qi Wang

**Affiliations:** ^1^Henan Neurodevelopment Engineering Research Center for Children, Henan Key Laboratory of Children's Genetics and Metabolic Diseases, Children's Hospital Affiliated to Zhengzhou University, Zhengzhou, China; ^2^Department of Rehabilitation, Children's Hospital Affiliated to Zhengzhou University, Zhengzhou, China; ^3^Department of Pathology, Children's Hospital Affiliated to Zhengzhou University, Zhengzhou, China; ^4^Center for Genetic Medicine, Xuzhou Maternity and Child Health Care Hospital, Xuzhou, China; ^5^Henan Infectious Disease Hospital, The Sixth People's Hospital of Zhengzhou, Zhengzhou, China; ^6^Department of Histology and Embryology, Guizhou Medical University, Guizhou, China

**Keywords:** autism spectrum disorder, metabotropic glutamate receptor 7, prefrontal cortex, action potential, mouse models

In the published article, there was an error in affiliation **1**. Instead of “Department of Nuclear Medicine, Affiliated Hospital of Guangdong Medical College, Zhanjiang, China,” it should be “Henan Neurodevelopment Engineering Research Center for Children, Henan Key Laboratory of Children's Genetics and Metabolic Diseases, Children's Hospital Affiliated to Zhengzhou University, Zhengzhou, China”.

The authors apologize for this error and state that this does not change the scientific conclusions of the article in any way. The original article has been updated.

## Publisher's Note

All claims expressed in this article are solely those of the authors and do not necessarily represent those of their affiliated organizations, or those of the publisher, the editors and the reviewers. Any product that may be evaluated in this article, or claim that may be made by its manufacturer, is not guaranteed or endorsed by the publisher.

