# Supplementary material for: Overexpression of mGluR7 in the Prefrontal Cortex Attenuates Autistic Behaviors in Mice
Source: Front Cell Neurosci. 2021 Jul 15;15:689611. doi: 10.3389/fncel.2021.689611 (PMC8319395; doi:10.3389/fncel.2021.689611)
Supplement: Supplementary file 1 [file Data_Sheet_1.PDF]

Supplementary Materials

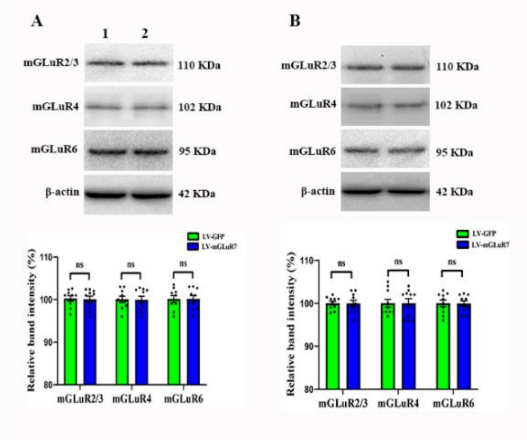

Figure S1 | Quantitative western blot analysis of mGluR2/3, mGluR4, and mGluR6 expression in the prefrontal cortex (PFC) samples of VPA-treated and BTBR mice. Representative western blot and the quantification of mGluR2/3, mGluR4, and mGluR6 protein levels in VPA-exposed **(A)** and BTBR **(B)** mice are shown. **(A)** Band 1, saline-treated mice; Band 2 VPA-exposed mice. **(B)** Band 1, C57B6L/J group; Band 2, BTBR group. Results are expressed as percentages of normalized mGluR2/3, mGluR4, and mGluR6 levels measured in controls, defined as 100%. β-actin was used as a loading control for the normalization of the mGluR2/3, mGluR4, and mGluR6 signals. $n = 6$ mice in each group. NS indicating no statistical significance. Student's *t*-test. Results are shown as mean ± SEM.

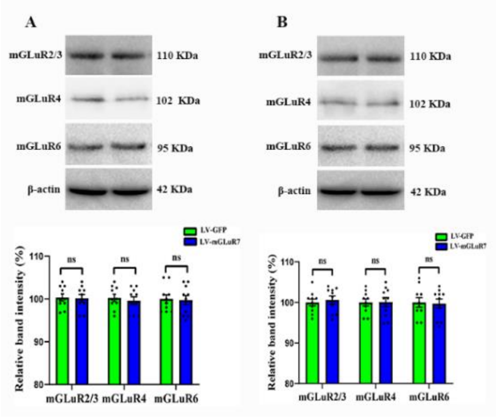

Figure S2 | Effects of mGluR7 overexpression on mGluR2/3, mGluR4, and mGluR6 levels in the PFC in animal models of ASD. Expression of mGluR2/3, mGluR4, and mGluR6 protein was significantly unaltered in VPA-treated **(A)** and BTBR mice **(B)**

overexpressing mGluR7 compared to mice treated with LV-GFP. Band 1, LV-mGluR7-treated group; Band 2, LV-GFP-exposed group. $n = 6$ mice per group. NS: no statistical significance. Student's *t*-test. Data are mean ± SEM.
